# Molecular signature induced by RNASET2, a tumor antagonizing gene, in ovarian cancer cells

**DOI:** 10.18632/oncotarget.274

**Published:** 2011-06-04

**Authors:** Francesco Acquati, Laura Monti, Marta Lualdi, Marco Fabbri, Maria Grazia Sacco, Laura Gribaldo, Roberto Taramelli

**Affiliations:** ^1^ Dipartimento di Biotecnologie e Scienze Molecolari, Università degli Studi dell'Insubria, via JH Dunant 3, 21100 Varese, Italy; ^2^ European Commission - Joint Research Centre Institute for Health and Consumer Protection Molecular Biology and Genomics unit TP 464, Via E. Fermi, 2749 21027 Ispra (VA) - Italy

**Keywords:** RNases, cancer microenvironment, transcriptional profile

## Abstract

Using the Hey3Met2 human ovarian cancer cell line, we previously found the *RNASET2* gene to possess a remarkable *in vivo* tumor suppressor activity, although no *in vitro* features such as inhibition of cell proliferation, clonogenic potential, impaired growth in soft agar and increase in apoptotic rate could be detected. This is reminiscent of the behavior of genes belonging to the class of tumor antagonizing genes (TAG) which act mainly within the context of the microenvironment. Here we present transcriptional profiles analysis which indicates that investigations of the mechanisms of TAG biological functions require a comparison between the *in vitro* and *in vivo* expression patterns. Indeed several genes displaying a biological function potentially related to tumor suppression could not be validated by subsequent *in vivo* expression analysis. On the other hand the fact that we could find congruency for three genes both in vivo and in vitro adds a warning to a too much stringent categorization of this class of genes which relies on the sensitivity of the methodological approaches.

## RNASET2, A NEW MEMBER OF THE GROWING CLASS OF TUMOR ANTAGONIZING GENES

Among the gynaecological malignancies, ovarian cancer is considered to be the most lethal tumor, with an incidence of 42,000 new cases per year in Europe [1] and 22,000 cases in the USA [2]. In more than 60% of the patients, the diagnosis is made in advanced stage, owing both to the lack of symptoms and the to fact that a panel of highly predictive biomarkers for screening and early stage detection is still missing. It is estimated that 80% of the patients with advanced disease will develop recurrence and succumb to the illness, despite their good initial responsiveness to primary therapy.

We reckon that one of the main reasons for such regrettable scenario is a poor understanding of the biological bases of ovarian cancer pathogenesis. Indeed, one of the salient feature of these tumors is their high heterogeneity, both at the morphological and biological levels, which make the contribution of genetic lesions difficult to interpret in a causative way. Among human cancers located in the ovary, those derived from the ovarian surface epithelium are the most frequent [3] and all epithelial ovarian cancer subtypes have been postulated to originate from the single layer of cells representing the ovary’s surface epithelium (OSE cells) [4]. These cells are known to undergo repeated cycles of proliferation due to the recurrent growth and rupture of ovarian follicles during the ovulatory cycle and one feature of this phenomenon is a well characterized interaction between the ovarian mesenchymal and surface epithelial cells [5]. Any imbalance in the microenviromental homeostasis has therefore the potential to contribute to ovarian cancerogenesis, and a growing interest has indeed been placed toward the active role for stromal misregulation in the progression of neoplasias [6]. Within this frame, a special class of genes has been recently described that seems to play a crucial role in tumor suppression by means of regulating the cross-talk between stromal and epithelial cells. These genes belong to the growing but still poorly characterized class of tumor antagonizing/ malignancy suppressor genes (TAG/MSG) [7], whose principal feature is their ability to suppress malignant growth *in vivo* but not *in vitro*.

We have recently reported a preliminary biological characterization of one of these genes, called RNASET2, which codes for an extracellular RNase highly conserved among the *phila* from viruses to humans, suggesting an evolutionary important function [8].

In a xenograph model for ovarian cancer, we found RNASET2 to carry out a strong oncosuppressive activity by recruiting cells of the monocyte/macrophage lineage into the tumour mass. This subset of cells represents the main component of the host immunological response [9]. Although the main function of RNASET2 was found to take place in the context of the microenvironment, namely in the extracellular compartment, we could not completely rule out that this conserved RNase might also carry out a cell-autonomous role, as suggested by a recent work on S.Cerevisiae [10].

This was basically the rationale that prompted us to further investigate the cell-autonomous expression profile induced by RNASET2 in ovarian cancer cells.

## *IN VITRO* WHOLE GENOME TRANSCRIPTIONAL PROFILING OF CONTROL AND RNASET2-OVEXPRESSING HUMAN OVARIAN CANCER CELLS

As previously reported, using the Hey3Met2 human ovarian cancer cell line, we found the *RNASET2* gene to possess a remarkable *in vivo* tumor suppressor activity, irrespective of the protein’s catalytic activity [9]. Noteworthy, when tested *in vitro*, the same cell clones did not show inhibition of cell proliferation, changes in the clonogenic potential, impaired growth in soft agar and increase in apoptotic rate.

As stated in the previous section, although these data strongly suggest that *RNASET2* might represent a new member of the family of TAG/MSG (acting mostly in a non-cell autonomous fashion), we could not formally rule out a cell-autonomous effect elicited by this gene on the cancer cells, which might have escaped detection by our panel of *in vitro* standard assays. We therefore decided to carry out a more thorough analysis to shed light on this issue.

Accordingly, we defined the expression profile of the *RNASET2*-overexpressing Hey3Met2 cells and compared it with that of control cells. An Agilent Whole Human Genome Oligo Microarray was employed with total RNA extracted from Hey3Met2 clones transfected with wild-type or a catalytically mutant form of RNASET2 (whose cDNA was mutagenized in the two CAS catalytic sites). Total RNA from Hey3Met2 cells, transfected with the empty vector, was used as a control.

**Figure 1:**
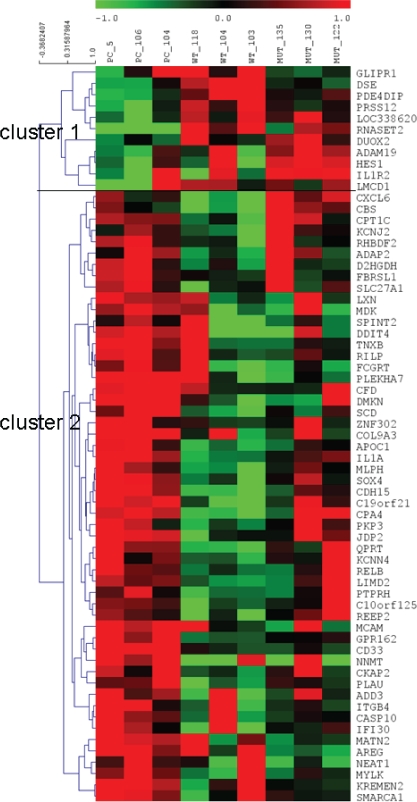
Heatmap of genes differentially expressed Modulated genes were organized in 2 clusters by hierarchical clustering. Each column corresponds to a single biological replicate. Each row represents a gene, with red and green for high and low expression levels, respectively.

Sixty-five genes were found to be modulated by at least one of the two RNASET2 protein forms tested (i.e. either wild-type or catalytically dead RNASET2), with a fold-induction or repression greater than 2 and a *p*-value smaller than 0.01. These genes were further organized by hierarchical clustering using squared Pearson correlation into 2 clusters, as shown in figure 1. Overall, for several of these genes we noticed a trend for a higher degree of change in their expression levels in Hey3met2 cells transfected with wild-type rather than catalytically-mutant *RNASET2* expression vectors. This might, hypothetically, suggest some influence of RNASET2 ribonucleolytic activity on the steady state level of these gene’s transcripts.

In order to define the cellular processes/pathways affected by RNASET2, all differentially expressed genes were cross-referenced to the Gene Ontology (GO) database to identify functional categories over-represented in the panel of genes associated with the two clusters.

The GO analysis was done at GO FAT level of biological process and molecular function, whereas Expression Analysis Systematic Explorer (EASE) biological theme analysis was carried out online (at http://david.niaid.nih.gov) using DAVID [11]. The GO survey did not show any significant enrichment, probably due to the small number of genes analyzed (54 genes in cluster 1 and 11 genes in cluster 2). Nevertheless, within the above mentioned gene set a few associated categories were shown to represent biologically relevant process that were worth investigating.

Since TAG/MSG genes are postulated to encode for products involved in the cross-talk between the cancer cell and the tumor microenvironment [12,13], we selected five *RNASET2*-responsive genes, mainly because of of their involvement in cell adhesion and migration. The five selected genes are *MDKN*, encoding an angiogenic growth factor over-expressed in various human malignant tumors and recently suggested as a new biomarker for ovarian cancer [14-16]; *AREG* (cell invasion breast cancer), a regulator of cancer cells invasiveness which was reported to be frequently overexpressed in colon, breast, prostate, pancreas, lung and ovarian cancer [17-19]; *MCAM*, a well known marker of poor prognosis in epithelial ovarian cancer, whose gene product promotes the growth, invasion and metastasic potential of malignant cells [20-22]; *PLAU* (PLasminogen Activator Urokinase-type), which promotes fibrinolysis and degradation of extracellular matrix [23]; and *NNMT* (nicotinamide N-methyltransferase), a serum tumor marker for colorectal cancer recently identified as novel regulator of cell migration [24,25].

Moreover, on the basis of their biological function we decided to validate a few genes that were also shown to be down-regulated after RNASET2 overexpression. The first is *DMKN*, a gene differentially regulated in inflammatory conditions, and whose role in activating Rab5 function was recently defined [26]. The genes encoding for the transcription factors RELB and JPD2 were also selected, due to their involvement in the NF-kB pathway and cell proliferation/differentiation, respectively [27-30].

The *DDIT4* gene was chosen due to its role in RAS-mediated transformation of ovarian epithelial cells [31]. Significantly, transfection of *DDIT4* expression vectors in immortalized human ovarian surface epithelial cells has been shown to render the cells tumorigenic in substitution for oncogenic Ras mutations.

Recent studies have suggested a relation between *CPA4* gene expression and prostate cancer aggressiveness, possibly due to its role in extracellular peptide processing [32]. We thus included *CPA4* in the panel of genes to be further investigated.

The *PTPRH* gene, encoding a member of the protein tyrosine phosphatase (PTP) family [33], was also selected, since PTP proteins are known to regulate a variety of cellular processes including cell growth, differentiation, mitotic cycle, and oncogenic transformation.

Finally, two genes that were up-regulated by *RNASET2* were also selected for validation: *LMCD1*, belonging to the category of the *LIMD1* tumor suppressor gene, and *DSE* (dermatan sulfate epimerase), whose protein product elicits specific cytotoxic T lymphocytes responses in cancer patients [34,35]. Table 1 provides a list of the thirteen genes selected for validation.

**Table 1:** List of RNASET2-modulated genes selected on the basis of fold change, GO analysis and biological relevance

GENE SYMBOL	GENE DESCRIPTION	FOLD-CHANGE WT	FOLD-CHANGE MUT	GENE ONTOLOGY
MDK	midkine (neurite growth promoting factor 2)	−2.5	−2.9	heparin binding; glycosaminoglycan binding
MCAM	melanoma cell adhesion molecule	−3	−2.8	cell adhesion;motility
AREG	amphiregulin	−2.1	−3.8	growth factor activity-cell invasion
NNMT	nicotinamide N-methyltransferase	−4.1	−5.1	cell migration
PLAU	plasminogen activator,urokinase	−2.0	−1.2	wound healing; fibrinolysis and degradation of extracellular matrix.
DDIT4	DNA-damage-inducible transcript 4	−4.3	−2.2	
CPA4	Carboxypeptidase A4	−3.8	−1.3	peptidase activity;zinc ion binding.
RELB	v-rel reticuloendotheliosis viral oncogene homolog B	−2.1	−1.8	DNA binding; transcription factor activity; transcription regulator activity
JDP2	Jun dimerization protein 2	−3.4	−2.4	DNA binding;transcription factor activity;transcription regulator activity
DMKN	dermokine	−6.7	−6.2	Rab GTPase binding
PTPRH	protein tyrosine phosphatase receptor type H	−2	−1.5	phosphoprotein phosphatase activity;transmembrane receptor protein phosphatase activity
DSE	dermatan sulfate epimerase	+2.1	+1.2	racemase and epimerase activity;chondroitin-glucuronate 5-epimerase activity
LIMCD1	LIM and cysteine-rich domains 1	+2.6	+2.3	zinc ion binding;transcription factor binding;transcription regulator activity

## VALIDATION AND COMPARISON OF THE TRANSCRIPTIONAL PROFILES *IN VITRO* AND *IN VIVO*

**Figure 2:**
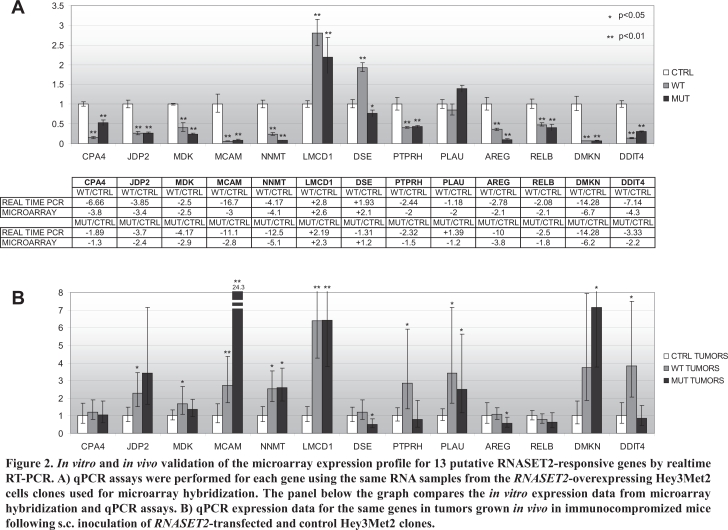
*In vitro* and *in vivo* validation of the microarray expression profile for 13 putative RNASET2-responsive genes by realtime RT-PCR A) qPCR assays were performed for each gene using the same RNA samples from the RNASET2-overexpressing Hey3Met2 cells clones used for microarray hybridization. The panel below the graph compares the in vitro expression data from microarray hybridization and qPCR assays. B) qPCR expression data for the same genes in tumors grown in vivo in immunocompromized mice following s.c. inoculation of RNASET2-transfected and control Hey3Met2 clones.

To validate these genes, realtime RT-PCR (qPCR) assays were performed on the same RNA samples used for microarray hybridization. In order to allow for cross-references of the expression data, the sequence of all primer pairs was designed to span the region of the cDNA corresponding to the hybridization probes placed on the microarray chip. As shown in figure 2A, the pattern of gene expression changes observed following microarray hybridization was confirmed by qPCR for most tested genes. The only exception was represented by the *DSE* and *PLAU* genes, whose expression pattern in Hey3Met2 cells expressing the *catalytically-mutant* RNASET2 protein turned out to be down-regulated by microarray hybridization but upregulated by qPCR. However, for both genes the observed change in expression pattern with respect to control clones was below the chosen 2-fold threshold for significance (+1.2-fold by microarray hybridization vs. −1.43-fold by qPCR for *DSE* and −1.2-fold by microarray hybridization vs. + 1.05-fold by qPCR for *PLAU*, respectively). Moreover, for both genes the expression pattern observed by qPCR in Hey3Met2 cells expressing *wild-type* RNASET2 was in agreement with that observed by microarray hybridization.

As expected for using a more sensitive test, the observed fold-changes in the expression levels appeared to be slightly higher for several genes when tested by qPCR. For example, the RNASET2-mediated downregulation of the *CPA4*, *DMKN* and *MCAM* genes that was observed by microarray hybridization turned out to be much more evident when assayed by qPCR. Of note, the fold-change expression for the *RNASET2* gene turned out to be much higher by qPCR, in keeping with previous expression data obtained by western blot analysis carried out on the same clones (data not shown). We next asked whether some of these genes could have any relevance in *RNASET2*-mediated tumor suppression *in vivo*. To this end, total RNA extracted from xenograph tumors [9] was used to assess whether the expression pattern was congruent with the qPCR data. Clearly at variance with the *in vitro* setting, the *in vivo* tumor population is rather heterogeneous, comprising both human Hey3Met2 cancer cells and host-derived murine stromal cells. The results of this survey are shown in figure 2B. As shown, the *RNASET2*-mediated changes in the expression levels that we previously observed *in vitro* were not confirmed for most tested genes in the *in vivo* setting. Indeed, significant changes in the expression pattern that were found to be in agreement with those previously observed *in vitro* could be reported for just three genes, namely *LMCD1*, *DSE* and *RELB*.

As for the remaining genes, whereas some of them (i.e. *CPA4*, *MDK* and *AREG*) are almost unresponsive to RNASET2 expression *in vivo*, others showed obvious changes in expression pattern mediated by RNASET2, but these changes were in the opposite direction with respect to those observed *in vitro*.

## FUTURE DIRECTIONS

Undoubtly, the observed *RNASET2*-mediated modulation of *LMCD1*, *DSE* and *RELB* gene expression deserves further investigation, since it has been detected *in vitro* and subsequently confirmed *in vivo*. These three genes thus represent *bona fide* candidate effector genes for *RNASET2*-mediated tumor suppression. A panel of ovarian cancer cell lines, characterized for *RNASET2* expression levels, is available in our laboratory and will be used to investigate the relationship between RNASET2 levels, expression of these three genes and putative phenotypic outcomes. Furthermore a *RNASET2*-knock-down model recently established in our laboratory in the OVCAR3 ovarian cancer cell line will further contribute to investigate this issue.

As for the putative mechanisms by which these three genes might contribute to *RNASET2*-mediated tumor suppression, it is worth noting that two of them show a plausible link with cellular functions related to tumor rejection by the immune system. Indeed, it is well established that one of the several biological functions of this pleiomorphic RNase is the modulation of host immune system [8].

Accordingly, the *RELB* gene has long been associated with adaptive immune responses mediated by dendritic cells [28,29]. Moreover, since *RELB* is directly involved in the NK-κB pathway, it is tempting to postulate a role for RNASET2 in *RELB*-mediated control of tumor-associated macrophages (TAM) polarization. In fact, tumor progression is thought to involve a gradual switch of macrophage polarization from M1 to M2 class, which is paralleled by a gradual inhibition of NK-κB activity [36]. This role for the NK-κB pathway in macrophage polarization might therefore be relevant, considering that *RNASET2* is apparently able to recruit specific subclass of stromal macrophages in the Hey3Met2 ovarian cancer model studied by our group [9]. It will be of interest in future studies to define whether RNASET2 derived from cancer cell, could influence, the NK-κB pathway by changing the *RELB* expression levels in cells of the monocyte/macrophage lineage. Moreover, due to the observed downregulation of *RELB* in Hey3Mey2 cells expressing *RNASET2*, a cell-autonomous role for *RNASET2* cannot be completely ruled out, since the RELB protein is known to promote cancer cell survival by inducing the expression of proteins with anti-apoptotic roles, such as Survivin and Bcl-2 [37]. A close inspection of the RELB yeast ortologue’ s behaviour in yeast cells could be very instructive in this regard.

The second *RNASET2*-responsive gene that might be related to immune cell function is *DSE*. It encodes for dermatan sulfate epimerase, an enzyme involved in D-glucuronic acid to L-iduronic acid conversion in the dermatan sulphate biosynthetic pathway [38]. The *DSE* gene was originally identified on the basis of the ability of its gene product to be recognized with high efficiency by a subset of cytotoxic T lymphocytes in certain tumors [35]. Moreover, expression of *DSE* was found to be altered in laryngeal cancer specimens when compared to normal tissue samples [39]. Significantly, abnormal dermatane sulphate composition has also been reported in both ovarian carcinomas and ovarian cancer cell lines [40], providing further support for a putative role of *DSE* in our ovarian model.

As for the third gene whose change in expression level was confirmed *in vivo* (*LMCD1*), its direct involvement in *RNASET2*-mediated tumor suppression is rather speculative. *LMCD1* encodes for a poorly characterized cysteine-rich and LIM domain-containing protein, which has been so far implicated in cardiac hypertrophy [41]. However, LIM-domain proteins belonging to the related CRP family have been described as potent tumor suppressor genes [42] and, most importantly, the related *LIMD1* gene has been recently reported to display functional properties reminiscent of tumor antagonizing genes [7].

Taken together, the results of our studies provides a clear indication that investigations of the molecular mechanisms through which TAG/MSG carry out their biological functions necessitate a thorough comparison between the *in vitro* and *in vivo* expression patterns. Indeed, several genes displaying a biological function potentially related to tumor suppression, and thus defined as candidate based on their *in vitro* expression pattern, were not validated by subsequent *in vivo* expression profiling.

On the other hand, the fact that the same changes in the expression levels of a few genes (i.e. *RELB, LIMD1* and *DSE*) were observed both in vitro and in vivo deserves some comments. t We rekon that the results gathered in the present report highlight some of the limitations inherent in the conceptual process of categorizing relevant biological processes. In our case, the strict categorization of tumor antagonizing genes as genes endorsed with an asymmetric behavior (i.e. suppressing tumorigenicity without affecting *in vitro* growth) was clearly dependent on the sensitivity of the methodological approach employed. In fact, using the highly sensitive microarray analysis some genes’ features were detected in a congruent fashion in both the *in vivo* and *in vitro*.

The three above mentioned genes are therefore worth to be investigated in depth, as they could represent not only relevant candidates for the carcinogenetic process taking place in our ovarian cancer model, but they could also be instrumental and paradigmatic of a more molecularly-oriented classification of the growing family of tumor antagonizing genes.

We reckon that the latter issue is not irrelevant in contributing some conceptual and experimental support for a more systematic search and characterization of tumor antagonizing genes. To this end, it is worth reminding that the genome of a cancer cell is mainly characterized by DNA losses and given the limited number of tumor suppressor genes identified and characterized so far, we are probably seeing just the “tip of the iceberg”, with a large fraction of genes (including TAGs) protecting against malignant growth yet undiscovered.
